# Trust in Healthcare during COVID-19 in Europe: vulnerable groups trust the least

**DOI:** 10.1007/s10389-022-01705-3

**Published:** 2022-03-24

**Authors:** Johannes Beller, Jürgen Schäfers, Jörg Haier, Siegfried Geyer, Jelena Epping

**Affiliations:** Hannover Medical School, Carl-Neuberg-Str. 1, 30625 Hannover, Germany

**Keywords:** Trust, Healthcare, COVID-19, Pandemic, Vulnerable groups, Unmet needs

## Abstract

**Aim:**

We examined predictors of trust in the healthcare system during the COVID-19 pandemic in 27 European countries.

**Subjects and methods:**

We used population-based data drawn from the Living, working and COVID-19 survey (*N* = 21,884, 52% female, ages 18 to 92 years) covering 27 European countries dated June and July 2020. Multilevel linear regression, linear regression, and regression-tree analyses were conducted.

**Results:**

We found that most participants tended to trust the healthcare system, although a substantial part could still be classified as distrusting (approx. 21%). Multiple variables, including being middle-aged or of older age, being female, lower levels of education, unemployment, worse general health status, having income difficulties, having unmet needs for healthcare, no healthcare contact during the COVID-19 pandemic, higher mental distress, and loneliness, were significantly associated with lower levels of trust. Among these variables mental distress, income difficulties, and unmet needs for healthcare emerged as especially important and, across European regions and countries, consistent predictors for lower trust in the healthcare system during the COVID-19 pandemic.

**Conclusions:**

Medically vulnerable subgroups, such as individuals with unmet healthcare needs, higher levels of mental distress, and older age, as well as people living in socially and economically vulnerable situations, such as higher levels of loneliness and financial difficulties, were the least trusting of the healthcare system during the COVID-19 pandemic. As these vulnerable subgroups are also at highest risk for contracting COVID-19 and experiencing negative COVID-19-related outcomes, more targeted prevention and intervention efforts should be implemented in these groups.

**Supplementary Information:**

The online version contains supplementary material available at 10.1007/s10389-022-01705-3.

## Background

Trust is fundamental to the functioning of healthcare (Rowe and Calnan 2006; Gille et al. 2015). Patients must trust the healthcare system to value their well-being and health above all other potential interests so that patients are willing to seek healthcare, accept diagnoses, and are compliant with treatments (Tucker et al. 2016; Lichter 2017). From an empirical perspective, trust in the healthcare system has been shown to predict health-related outcomes such as health-related quality of life, patient satisfaction, therapy compliance, as well as disease-related objective parameters such as CD4 cell counts and glycaemic control (Armstrong et al. 2006; Kelley et al. 2014; Birkhäuer et al. 2017; Lee et al. 2020). Accordingly, promoting trust and achieving acceptance are embedded in the ethical guidelines of healthcare practice and are considered key criteria of universal health coverage (General Medical Council 2013; Khullar 2019).

Maintaining trust in the healthcare system seems especially important during pandemics, such as the current one caused by SARS-CoV-2 (Vinck et al. 2019; Udow-Phillips and Lantz 2020; Balog-Way and McComas 2020; Bekker et al. 2020; Wong et al. 2021). Prevention and control of pandemics rely on wide-ranging freedom-restricting public health interventions, such as shut-downs and stay-at-home orders, as well as compliance with medical interventions, such as vaccinations. Support and compliance for these interventions depends on trust (Chan et al. 2008, 2020). On the other hand, reduced capacities for regular healthcare, rising medical uncertainty, occurrence of decision conflicts, and changing treatment algorithms due to the pandemic constitute clear threats to public trust in the healthcare system (Armstrong and Freiberg 2017; Udow-Phillips and Lantz 2020). However, perception of these changes seems to differ between various population groups and regions (e.g., Sloan et al. 2021; Eder et al. 2021; Masters et al. 2021; Büssing et al. 2021; Lindholt et al. 2021; Beller et al. 2021). Empirically, several predictors have been found to predict trust in the health care system during routine care, including previous contact with the health care system, mental health, socio-economic status, urbanicity, and one’s general health status (Guerrero et al. 2015; Zhao et al. 2019a, b). Additionally, having unmet needs for healthcare might be another predictor that is especially important during the COVID-19 pandemic. However, studies are lacking that empirically examine predictors of trust in the healthcare system during pandemics.

Therefore, there is a need to study predictors of trust in the healthcare system during pandemics such as COVID-19 and to identify subgroups who are the least trusting. The current study aims to address this issue. It contributes to the existing literature by examining predictors of trust in healthcare during the COVID-19 pandemic in 27 European countries (Austria, Belgium, Bulgaria, Croatia, Cyprus, Czechia, Denmark, Estonia, Finland, France, Germany, Greece, Hungary, Ireland, Italy, Latvia, Lithuania, Luxembourg, Malta, Netherlands, Poland, Portugal, Romania, Slovakia, Slovenia, Spain, and Sweden).

## Methods

### Sample

Cross-sectional data from the Living, working and COVID-19 survey (second round) were used for the current study (Eurofound 2020). Consistently across countries, data were collected via an online survey in June and July 2020 by the European Foundation for the Improvement of Living and Working Conditions. This involved a snowballing process using mailing lists and advertising on social media platforms such as Facebook and Twitter to survey people aged 18 and older living in Europe to investigate how Europeans are dealing with the COVID-19 pandemic. Specifically, participants were recruited via publishing the link to the survey on Eurofound’s websites, mailing lists, and social media platforms. Additionally, Eurofound’s contacts and stakeholders were asked to further share the link via their websites, mailing lists, and social media platforms to reach as many participants as possible. Thus, only participants with access to the internet could take part in the survey. As in other non-probabilistic online surveys, the elderly, people living in rural areas, and people with a low educational level were thus less likely to participate in this study, and the survey cannot be taken to be fully representative of the populations in Europe (European Foundation for the Improvement of Living and Working Conditions 2020; European Foundation for the Improvement of Living and Working Conditions. 2021). Thematically, the questionnaire collected information related to living and working conditions, well-being, and use of public services during the COVID-19 pandemic. The survey drew on validated operationalizations of variables previously tested in the European Quality of Life Survey and European Working Conditions Survey (European Foundation for the Improvement of Living and Working Conditions. 2017a, b; European Foundation for the Improvement of Living and Working Conditions 2020). However, no formal psychometric tests on the new online questionnaire have been conducted. The questionnaire was developed in English but also translated in 21 additional languages: Bulgarian, Croatian, Czech, Danish, Dutch, Estonian, Finnish, French, German, Greek, Hungarian, Italian, Latvian, Lithuanian, Polish, Portuguese, Romanian, Slovak, Slovene, Spanish, and Swedish. The questionnaire can be accessed online (https://www.eurofound.europa.eu/sites/default/files/wpef20023.pdf). These data were provided to the principal author of the current study upon a confidentiality agreement. All in all, 24,123 participants were recruited, of which 91% fully completed the relevant questions. After deleting participants with missing values, a final sample size of *N* = 21,884 resulted: (*N*_*Austria*_ = 736; *N*_*Belgium*_ = 831; *N*_*Bulgaria*_ = 920; *N*_*Croatia*_ = 945; *N*_*Cyprus*_ = 342; *N*_*Czechia*_ = 596; *N*_*Denmark*_ = 629; *N*_*Estonia*_ = 531; *N*_*Finland*_ = 581; *N*_*France*_ = 616; *N*_*Germany*_ = 1043; *N*_*Greece*_ = 950; *N*_*Hungary*_ = 1481; *N*_*Ireland*_ = 2218; *N*_*Italy*_ = 933; *N*_*Latvia*_ = 472; *N*_*Lithuania*_ = 902; *N*_*Luxembourg*_ = 320; *N*_*Malta*_ = 365; *N*_*Netherlands*_ = 486; *N*_*Poland*_ = 557; *N*_*Portgual*_ = 1542; *N*_*Romania*_ = 1201; *N*_*Slovakia*_ = 660; *N*_*Slovenia*_ = 427; *N*_*Spain*_ = 1099; *N*_*Sweden*_ = 501).

### Measures

Trust in the healthcare system as our outcome was measured by asking “Please answer on a scale of 1–10 how much you personally trust each of the following institutions: The healthcare system” and operationalized on a scale from “Do not trust at all” [1] to “Trust completely” [10]. Predictor variables were age, gender, education (coded as “Non-tertiary” [0], “Tertiary” [1]; education was originally collected in the questionnaire via the three categories of primary education, secondary education, and tertiary education. As primary education levels were very low in some countries, the categories needed to be collapsed), employment (coded as “Not employed” [0], “Employed” [1]), health status (coded as “Not good” [0], “Good” [1]), income difficulties (coded as “No difficulties” [0], “Difficulties” [1]), unmet needs for healthcare (by asking “Since the pandemic began, have you needed a medical examination or treatment that you have not received?” with answers coded as “No unmet medical care need” [0] and “Unmet medical care need” [1]), healthcare contact (coded as “No contact” [0], “Had contact during pandemic” [1]), mental distress (operationalized via the WHO-5 cut-off score, coded as “No distress” [0], “Distress” [1]), and loneliness (coded as “Not Lonely More Than Half the Time” [0], “Lonely More Than Half the Time” [1]). Countries were furthermore classified as belonging to European regions (North: Denmark, Estonia, Finland, Ireland, Lithuania, Latvia, Sweden; West: Austria, Belgium, Germany, France, Luxembourg, Netherlands; East: Bulgaria, Czechia, Hungary, Poland, Romania, Slovakia; South: Croatia, Cyprus, Greece, Italy, Malta, Portugal, Slovenia, Spain) based on United Nations geo-scheme (United Nations 2021).

### Data analysis

First, the data were analyzed descriptively. As an inferential statistical analysis, multilevel linear regression with a varying intercept per country was performed for all participants and stratified by European regions with trust as the criterion and the other variables as predictors. Trust in healthcare has been shown to be associated with a wide range of factors in the literature; accordingly, using a regression-based approach enabled us to examine the potential associations of our multiple predictors of trust in healthcare simultaneously. To analyze the robustness of the results across countries, linear regression analysis with the same set of variables was additionally performed for each country. As a further robustness analysis, the method of *p* value based regression tree analysis was additionally applied. Here, as a method of recursive partitioning, subgroups as homogeneous as possible with respect to the criterion and with a maximum depth of three splits were formed on the basis of the predictors using non-parametric permutation tests. Given that the sample was obtained using a non-probability sampling methodology, the composition of the sample can be adjusted to make it more representative of the European populations. Hence, all descriptive and regression analyses are weighted with the weights provided with the data set so that the sample is statistically matched to the European population regarding gender, age, education, and self-defined urbanization levels (European Foundation for the Improvement of Living and Working Conditions 2020). All analyses were performed using R.

## Role of the funding source

The German Federal Ministry of Education and Research (BMBF) had no role in the study design, in the collection, analysis, and interpretation of data, in the writing of the report, and in the decision to submit the paper for publication.

## Results

Weighted descriptive statistics are displayed in Table 1. Participants were on average 49.40 years old (Standard Deviation [*SD*] = 16.58; Range = 18–92), with 52% being female. Approximately 29% of participants reported a tertiary level of education. Furthermore, most of participants reported no difficulties with their current income (57%), were employed (51%), had a good health status (62%) and no unmet needs for healthcare (79%). Furthermore, 67% of participants reported previous contact with healthcare providers during the pandemic, and the minority of participants were classified with having mental distress (42%) or being lonely (20%). Finally, an average level of trust in healthcare was observed (*M* = 6.53, *SD* = 2.46). Low trust in the health care system (score < = 4) was seen in approximately 21% of participants, whereas approximately 20% reported intermediate trust (score = 5 or 6), and approximately 59% high trust (score > = 7). Descriptive statistics were generally similar across European geographical regions (Table 1). However, participants in Eastern Europe tended to report much less trust in the healthcare system as compared with other European regions, whereas unmet needs for healthcare seemed especially low in Western Europe during the pandemic.

**Table 1:** Descriptive statistics of trust in the healthcare system and its predictors during the COVID-19 pandemic in Europe (*N* = 21,884)

	All (*N* = 21,884)		Northern (*N* = 5834)		Western (*N* = 4032)		Eastern (*N* = 5415)		Southern (*N* = 6603)	
	M / %	SD	M / %	SD	M / %	SD	M / %	SD	M / %	SD
Trust in healthcare	6.53	2.46	6.98	2.32	6.97	2.24	4.68	2.53	7.06	2.11
Low level of trust (score < = 4)	21%		16%		14%		49%		12%	
Average level of trust (score = 5 or 6)	20%		18%		20%		24%		19%	
High level of trust (score > = 7)	59%		66%		66%		27%		68%	
Age	49.40	16.58	48.95	16.83	49.70	17.01	48.14	16.07	49.93	16.25
Gender (female)	52%	–	49%	–	52%	–	52%	–	52%	–
Education (tertiary)	29%	–	39%	–	31%	–	25%	–	26%	–
Having income difficulties	43%	–	32%	–	38%	–	53%	–	44%	–
Being employed	51%	–	56%	–	50%	–	54%	–	49%	–
Health status (good)	62%	–	57%	–	65%	–	54%	–	66%	–
Unmet healthcare needs)	21%	–	21%	–	16%	–	27%	–	24%	–
Had contact to healthcare system during the COVID-19 pandemic	67%	–	63%	–	67%	–	64%	–	69%	–
Reporting mental distress	42%	–	39%	–	38%	–	46%	–	44%	–
Reporting loneliness	20%	–	22%	–	21%	–	21%	–	19%	–

Next, we identified predictors for trust in the healthcare system by using multilevel regression, as depicted in Table 2. It was found that all investigated variables significantly predicted trust overall, including age group, with middle-aged adults exhibiting the lowest predicted trust, gender, educational level, income difficulties, employment, health status, healthcare contact, unmet healthcare needs, mental distress, and loneliness. The strongest effect sizes, however, were exhibited by unmet healthcare needs, with reporting unmet health needs being strongly associated with a predicted decrease in trust as visible in the adjusted regression coefficient of *b* = −0.72. Furthermore, mental distress and income difficulties are also shown to be associated with a strong decrease of trust, with adjusted regression coefficients of *b* = −0.59 and *b* = −0.51, respectively.

**Table 2:** Results from multilevel linear regression predicting trust in the healthcare system during the COVID-19 pandemic in Europe (N = 21,884)

		All			Northern Europe			Western Europe			Eastern Europe			Southern Europe	
	*B*	*95%-CI*	*p*	*b*	*95%-CI*	*p*	*b*	*95%-CI*	*p*	*b*	*95%-CI*	*p*	*b*	*95%-CI*	*p*
Age group															
Age group 18–39 (Ref.)															
Age group 40–65	−0.39	(−0.46, −0.33)	< .001	−0.35	(−0.48, −0.22)	< .001	−0.42	(−0.58, −0.26)	< .001	−0.52	(−0.66, −0.38)	< .001	−0.22	(−0.33, −0.10)	< .001
Age group 66+	−0.21	(−0.30, −0.12)	< .001	−0.24	(−0.42, −0.07)	.006	−0.21	(−0.42, 0.00)	.049	−0.60	(−0.80, −0.41)	< .001	0.08	(−0.07, 0.23)	.302
Gender															
Male (Ref.)															
Female	−0.06	(−0.12, 0.00)	.041	0.04	(−0.07, 0.15)	.429	−0.06	(−0.20, 0.07)	.363	−0.10	(−0.22, 0.03)	.134	−0.07	(−0.16, 0.02)	.146
Education															
Non-tertiary education (Ref.)															
Tertiary education	0.15	(0.09, 0.22)	< .001	−0.05	(−0.17, 0.07)	.406	0.41	(0.26, 0.56)	< .001	0.09	(−0.05, 0.24)	.206	−0.10	(−0.22, 0.01)	.066
Income difficulties															
No Difficulties to cope (Ref.)															
Difficulties to cope	−0.51	(−0.57, −0.45)	< .001	−0.50	(−0.63, −0.37)	< .001	−0.61	(−0.76, −0.46)	< .001	−0.48	(−0.61, −0.34)	< .001	−0.39	(−0.49, −0.29)	< .001
Employment															
Not exployed (Ref.)															
Empoyed	−0.07	(−0.14, −0.01)	.029	0.04	(−0.09, 0.17)	.578	−0.08	(−0.24, 0.07)	.295	−0.25	(−0.39, −0.10)	< .001	−0.01	(−0.12, 0.10)	.871
Health status															
Not Good (Ref.)															
Good	0.18	(0.12, 0.25)	< .001	0.38	(0.25, 0.51)	< .001	0.07	(−0.08, 0.22)	.363	0.43	(0.30, 0.57)	< .001	0.08	(−0.03, 0.18)	.172
Healthcare contact															
No Contact (Ref.)															
Contact	0.41	(0.35, 0.47)	< .001	0.26	(0.15, 0.38)	< .001	0.32	(0.18, 0.47)	< .001	0.56	(0.43, 0.70)	< .001	0.46	(0.36, 0.57)	< .001
Healthcare needs															
No Unmet Healthcare Needs (Ref.)															
Unmet Healthcare Needs	−0.72	(−0.79, −0.65)	< .001	−0.77	(−0.91, −0.63)	< .001	−0.93	(−1.11, −0.74)	< .001	−0.37	(−0.52, −0.23)	< .001	−0.74	(−0.85, −0.63)	< .001
Mental distress															
No Distress (Ref.)															
Distress	−0.59	(−0.65, −0.52)	< .001	−0.75	(−0.88, −0.61)	< .001	−0.41	(−0.57, −0.25)	< .001	−0.60	(−0.74, −0.46)	< .001	−0.75	(−0.85, −0.64)	< .001
Loneliness															
Not Lonely (Ref.)															
Lonely	−0.21	(−0.29, −0.13)	< .001	−0.26	(−0.41, −0.12)	< .001	−0.31	(−0.48, −0.13)	< .001	−0.24	(−0.41, −0.08)	.004	−0.02	(−0.15, 0.10)	.701

These findings were mostly replicated across all European geographical regions (Table 2). However, having unmet needs for healthcare seemed especially detrimental to trust in Western Europe (*b* = −0.92) but not as detrimental in Eastern Europe (*b* = −0.37). Additionally, belonging to the age group of older adults and loneliness were not significantly associated with decreased trust in Southern Europe, in contrast to the other regions. These results were also confirmed by investigating predictors of trust for each country separately, as visualized in Fig. A1.

**Fig. 1:**
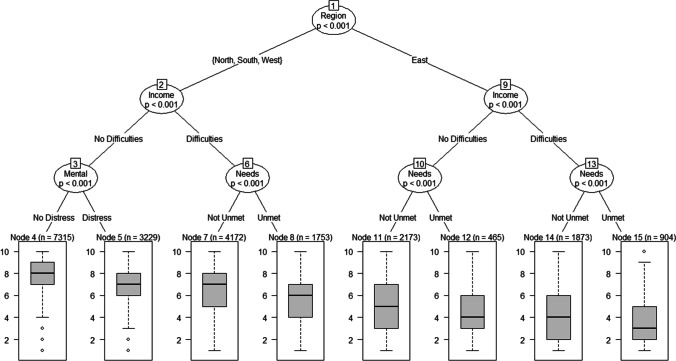
Results from the regression tree analysis predicting trust in the healthcare system during the COVID-19 pandemic

Finally, we identified subgroups regarding different levels of trust in the healthcare system during the occurrence of COVID-19 by using *p* value based regression tree analysis (Fig. 1). In accordance with the above-mentioned results, income difficulties, unmet healthcare needs, and mental distress proved to be among the most important determinants for predicting trust in the healthcare system. Again, in accordance with the above-mentioned results, similar distinctions between regions occurred, although, visible as the first split, participants in Eastern Europe exhibited much lower trust than participants from other regions. The subgroup with the lowest trust in the healthcare system was identified as the one from Eastern Europe who reported income difficulties and unmet needs for healthcare with an average trust in healthcare of only *M* = 3.41. In contrast, the highest trust was reported among participants from Northern, Southern, and Western Europe who reported no income difficulties and exhibited no mental distress with an average trust in the healthcare system of *M* = 7.45.

## Discussion

We examined predictors of trust in the healthcare system during the COVID-19 pandemic using a large cross-national sample of Europeans (*N* = 21,884) and found that most participants tended to trust their healthcare system in the investigated cohort. However, a substantial part of the included population can still be classified as distrusting (approx. 21% of the whole sample). Multiple variables, including age, gender, education, employment, health status, income difficulties, unmet needs for healthcare, healthcare contact, mental distress, and loneliness, were significantly and in a largely consistent way associated with a perception of trust of their healthcare system under the specific conditions of the pandemic (as an exemption to this statement, it has to be noted that tertiary education predicted higher trust for participants from Western Europe). Among these variables mental distress, income difficulties and unmet needs for healthcare emerged as especially important and consistent predictors for trust in the healthcare system during the COVID-19 pandemic, even across countries with larger out-of-pocket health care expenses (such as Bulgaria and Greece) and smaller out-of-pocket health care expenses (such as France and Germany). Mental distress, income difficulties, and unmet needs for healthcare also determined the most-distrusting subgroups across regions in the current study.

Our results both confirm and go beyond previous studies. As shown in our study, mental distress and (lack of) contact with the healthcare system have been reported to be among the strongest predictors of trust in the healthcare system (Guerrero et al. 2015; Zhao et al. 2019a, b). However, in contrast to some of the previous literature, we found that some socio-demographic predictors were significant in explaining the amount of trust in the healthcare system to a certain extent, albeit to a smaller degree than mental distress and unmet healthcare needs. Among these socio-demographic variables, income difficulties appeared to be the major predictor of participants’ trust in the healthcare system.

Going beyond the previously published literature the effects of the predictors on trust in the healthcare system occurred in a generalized manner and were found similarly across European regions and single countries, with few exceptions. The effect size of unmet needs for healthcare was especially large in Western Europe. This might be due to expectations in the populations of Western Europe that healthcare is always and universally available and accessible in a patient-cenered way (Coulter and Jenkinson 2005). When this expectation is not met, and the perception is such of a larger gap in health care delivery—at least for certain patient groups and regions during the COVID-19 pandemic—loss of trust might particularly impact participants from Western Europe (Gillespie and Dietz 2009). In contrast, unmet needs for healthcare appear to be a less important factor to trust in healthcare for participants from Eastern Europe. One explanation might be that in these countries, expectations of unhindered access to healthcare are generally not as high as in Western Europe (Cylus and Papanicolas 2015). Coming from a lower level of trust, limited universal access to health care during a pandemic is likely to have less impact on trust in health care for participants from these regions, as observed in our results. Finally, in contrast to other regions, loneliness was not significantly associated with reduced trust in the healthcare system in Southern Europe. One hypothesis as to why this occured might be the cultural differences (Carr 2019; Beller and Wagner 2020): Differences in the social behavior, such as the role of families as social environment and different cultural levels of collectivism might be possible explanations as to why the effects of loneliness on trust might be smaller in Southern European countries. Notwithstanding these small variations, the results were very consistent across regions and robust across different methods of analysis.

Therefore, to maintain functioning of the healthcare system in pandemics it seems important to address three central issues from the perspective of public health and public trust: First, the occurrence of unmet needs for healthcare needs to be minimized. This obviously is particular challenging during a pandemic and requires careful planning and adaptation of treatment algorithms as well as raising general health literacy (Ivanyi et al. 2021). Additionally, management of the population’s expectations regarding possible limitations in universally available healthcare as well as possibly reduced applicability of usual delivery processes is necessary, for example by targeted information campaigns (Harris 2017). Second, economic aid should be generously granted in order to relieve the financial distress, which, in turn, could lead to a corresponding increase in trust (Leibrecht and Scharler 2020). Third, mental health problems need dedicated attention, especially due to the severe impact on typical treatment mental health treatment algorithms during pandemics (Pfefferbaum and North 2020). When all of the above are being taken into account, the trust in the healthcare system during a pandemic can be maintained and, in turn, compliance of the population with healthcare mechanisms (such as wearing face masks in public, signing up for vaccination, and adhering to hygiene and distance rules) might be increased, which should be addressed in future studies.

## Limitations

When interpreting the results, several limitations of the analysis have to be taken into account. Most importantly, using a non-probabilistic, non-standardized sampling approach enabled the survey to quickly capture the potential impact of the COVID-19 pandemic in Europe. However, as the sample was recruited online via a snow-balling process, it cannot be considered as fully representative of the populations in Europe (Cornesse and Bosnjak 2018; MacInnis et al. 2018; Cornesse et al. 2020; Lehdonvirta et al. 2021). In Europe, a large part of the population has access to the internet, but many important sub-populations such as the elderly, people in rural areas, and people with a low educational level are thus less likely to participate in the survey (Lengsfeld 2011). Additionally, average levels of access to the internet vary in Europe, with Luxembourg and Sweden having comparatively high levels of access and Romania and Bulgaria having relatively low levels of access, and therefore a stronger selection bias might be expected to occur in some countries (Cruz-Jesus et al. 2016). Furthermore, in the literature, a recent study has shown that participation in an online survey was associated with behavioral patterns relating to COVID-19 and health in general has been found to be predictive of survey participation (Schaurer and Weiß 2020; Beller et al. 2022). As such, the sample is likely to be over-representative of more educated, younger, and healthier Europeans. Nonetheless, using this sampling strategy enabled the recruitment and analysis of a pan-European sample early in the COVID-19 pandemic.

Additionally, the questionnaire needed to be relatively short to collect an adequate sample size. As such, some constructs could only be measured with abbreviated items. For example, education was measured by asking participants about their completed educational level regarding few categories; it would have been beneficial to have access to more comprehensive operationalizations, which was not possible for all constructs, given the constraints of the questionnaire. In a similar vein, although the survey drew on validated operationalizations of variables, no formal psychometric tests on the new questionnaire have been conducted. Therefore, although several previous studies also did not find significant associations of educational level and trust in healthcare, it cannot be ruled out that lack of variance in education might be partly responsible for the observed lack of a significant relationship (Guerrero et al. 2015; Hong et al. 2018). We also could only use one item to measure trust in the healthcare system. However, trust has been conceptualized as a multidimensional phenomenon that compromises several levels (Fulmer and Dirks 2018). Correspondingly, more comprehensive multi-item instruments should be employed by future studies. Thus, although the sample size is large, the sample covers multiple European countries, and we found robust results across regions and countries, generalizability of the obtained results to other population-based samples and other operationalisations of trust needs to be addressed in future studies.

## Conclusions

There is a growing body of evidence concerning the public health importance of maintaining trust in healthcare, especially in pandemics, but it remained unclear which factors predict decreased trust in healthcare during pandemics. Using a cross-national European sample, we found high trust levels for most participants during the first wave of the COVID-19 pandemic, except in Eastern Europe. Additionally, we identified multiple variables, including age, gender, education, employment, health status, income difficulties, unmet needs for healthcare, healthcare contact during the COVID-19 pandemic, mental distress, and loneliness, that were significantly associated with trust in a mostly consistent way across countries and regions. These results suggest that medically vulnerable subgroups, such as individuals with unmet healthcare needs and more mental distress, and persons living in socially and economically vulnerable situations, such as higher levels of loneliness, and with financial difficulties, were least trusting in the healthcare system during the COVID-19 pandemic. As the identified factors are also characteristic of vulnerable subgroups who are highest at risk for contracting COVID-19 and experiencing negative COVID-19-related outcomes, more targeted prevention and intervention efforts should be implemented targeting these groups (Harris and Sandal 2021).

## Supplementary information

ESM 1 (DOCX 212 kb)

## Data Availability

The data that support the findings of this study are available from the European Foundation for the Improvement of Living and Working Conditions. Restrictions apply to the availability of these data, which were used under license for this study. Data are available from https://www.eurofound.europa.eu/data/covid-19 with the permission of the European Foundation for the Improvement of Living and Working Conditions.

## Code availability

N/A

## Declarations

### Data sharing

The data that support the findings of this study are available from the European Foundation for the Improvement of Living and Working Conditions. Restrictions apply to the availability of these data, which were used under license for this study. Data are available from https://www.eurofound.europa.eu/data/covid-19 with the permission of the European Foundation for the Improvement of Living and Working Conditions.

## References

[CR1] Armstrong K, Freiberg AA (2017). Challenges and opportunities in disclosing financial interests to patients. JAMA.

[CR2] Armstrong K, Rose A, Peters N (2006). Distrust of the health care system and self-reported health in the United States. J Gen Intern Med.

[CR3] Balog-Way DHP, McComas KA (2020). COVID-19: reflections on trust, tradeoffs, and preparedness. J Risk Research.

[CR4] Bekker M, Ivankovic D, Biermann O (2020). Early lessons from COVID-19 response and shifts in authority: public trust, policy legitimacy and political inclusion. Eur J Pub Health.

[CR5] Beller J, Wagner A (2020) Loneliness and health: the moderating effect of cross-cultural individualism/collectivism. J Aging Health 089826432094333. 10.1177/0898264320943336 10.1177/0898264320943336 32723203

[CR6] Beller J, Schäfers J, Geyer S (2021). Patterns of changes in oncological care due to COVID-19: results of a survey of oncological nurses and physicians from the region of Hanover. Germany Healthcare.

[CR7] Beller J, Geyer S, Epping J (2022). Health and study dropout: health aspects differentially predict attrition. BMC Med Res Methodol.

[CR8] Birkhäuer J, Gaab J, Kossowsky J (2017). Trust in the health care professional and health outcome: a meta-analysis. PLoS One.

[CR9] Büssing A, Recchia DR, Hübner J (2021). Tumor patients’ fears and worries and perceived changes of specific attitudes, perceptions and behaviors due to the COVID-19 pandemic are still relevant. J Cancer Res Clin Oncol.

[CR10] Carr D (2019). Aging alone? International perspectives on social integration and isolation. The J Gerontol: Series B.

[CR11] Chan DCC, Lee WTK, Lo DHS (2008). Relationship between grip strength and bone mineral density in healthy Hong Kong adolescents. Osteoporos Int.

[CR12] Chan HF, Brumpton M, Macintyre A (2020). How confidence in health care systems affects mobility and compliance during the COVID-19 pandemic. PLoS One.

[CR13] Cornesse C, Bosnjak M (2018). Is there an association between survey characteristics and representativeness? A meta-analysis. Survey Res Methods.

[CR14] Cornesse C, Blom AG, Dutwin D (2020). A review of conceptual approaches and empirical evidence on probability and nonprobability sample survey research. J Survey Statistics Methodol.

[CR15] Coulter A, Jenkinson C (2005). European patients’ views on the responsiveness of health systems and healthcare providers. Eur J Pub Health.

[CR16] Cruz-Jesus F, Vicente MR, Bacao F, Oliveira T (2016). The education-related digital divide: an analysis for the EU-28. Comput Hum Behav.

[CR17] Cylus J, Papanicolas I (2015). An analysis of perceived access to health care in Europe: how universal is universal coverage?. Health Policy.

[CR18] Eder SJ, Steyrl D, Stefanczyk MM (2021). Predicting fear and perceived health during the COVID-19 pandemic using machine learning: a cross-national longitudinal study. PLoS One.

[CR19] Eurofound (2020) Living, working and COVID-19 dataset. Dublin

[CR20] European Foundation for the Improvement of Living and Working Conditions (2017). European quality of life survey 2016 :quality of life, quality of public services, and quality of society : overview report.

[CR21] European Foundation for the Improvement of Living and Working Conditions (2017). 6th European working conditions survey: 2017 update.

[CR22] European Foundation for the Improvement of Living and Working Conditions (2020). Living, working and COVID-19.

[CR23] European Foundation for the Improvement of Living and Working Conditions (2021). Living, working and COVID-19 (update April 2021): mental health and trust decline across EU as pandemic enters another year.

[CR24] Fulmer A, Dirks K (2018). Multilevel trust: a theoretical and practical imperative. J Trust Res.

[CR25] General Medical Council (2013) Good Medical Practice

[CR26] Gille F, Smith S, Mays N (2015). Why public trust in health care systems matters and deserves greater research attention. J Health Serv Res Policy.

[CR27] Gillespie N, Dietz G (2009). Trust repair after an organization-level failure. AMR.

[CR28] Guerrero N, Mendes de Leon CF, Evans DA, Jacobs EA (2015). Determinants of trust in health care in an older population. J Am Geriatr Soc.

[CR29] Harris M (2017). Managing expense and expectation in a treatment revolution: Problematizing prioritisation through an exploration of hepatitis C treatment ‘benefit’. Int J Drug Policy.

[CR30] Harris SM, Sandal GM (2021). COVID-19 and psychological distress in Norway: the role of trust in the healthcare system. Scand J Public Health.

[CR31] Hong HC, Lee H, Collins EG (2018). Factors affecting trust in healthcare among middle-aged to older Korean American women. BMC Womens Health.

[CR32] Ivanyi P, Park-Simon T, Christiansen H et al (2021) Protective measures for patients with advanced cancer during the Sars-CoV-2 pandemic: quo vadis? Clin Exp Metastasis. 10.1007/s10585-021-10083-1 10.1007/s10585-021-10083-1 PMC7987238 33759009

[CR33] Kelley JM, Kraft-Todd G, Schapira L (2014). The influence of the patient-clinician relationship on healthcare outcomes: a systematic review and Meta-analysis of randomized controlled trials. PLoS One.

[CR34] Khullar D (2019). Building Trust in Health Care—why, where, and how. JAMA.

[CR35] Lee J, Lau S, Meijer E, Hu P (2020). Living longer, with or without disability? A global and longitudinal perspective. The Journals of Gerontology: Series A.

[CR36] Lehdonvirta V, Oksanen A, Räsänen P, Blank G (2021). Social media, web, and panel surveys: using non-probability samples in social and policy research. Policy Internet.

[CR37] Leibrecht M, Scharler J (2020) Trust dynamics after financial distress: evidence from euro member countries. Appl Econ Lett 1–6. 10.1080/13504851.2020.1855308

[CR38] Lengsfeld JHB (2011). An econometric analysis of the sociodemographic topology of the digital divide in Europe. Inf Soc.

[CR39] Lichter AS (2017). Conflict of interest and the integrity of the medical profession. JAMA.

[CR40] Lindholt MF, Jørgensen F, Bor A, Petersen MB (2021). Public acceptance of COVID-19 vaccines: cross-national evidence on levels and individual-level predictors using observational data. BMJ Open.

[CR41] MacInnis B, Krosnick JA, Ho AS, Cho M-J (2018). The accuracy of measurements with probability and nonprobability survey samples: replication and extension. Public Opin Q.

[CR42] Masters GA, Asipenko E, Bergman AL (2021). Impact of the COVID-19 pandemic on mental health, access to care, and health disparities in the perinatal period. J Psychiatr Res.

[CR43] Pfefferbaum B, North CS (2020). Mental health and the Covid-19 pandemic. N Engl J Med.

[CR44] Rowe R, Calnan M (2006). Trust relations in health care—the new agenda. Eur J Pub Health.

[CR45] Schaurer I, Weiß B (2020) Investigating selection bias of online surveys on coronavirus-related behavioral outcomes: an example utilizing the GESIS panel special survey on the coronavirus SARS-CoV-2 outbreak in Germany. Survey Res Methods 103-108 pages. 10.18148/SRM/2020.V14I2.7751

[CR46] Sloan M, Gordon C, Harwood R (2021). The impact of the COVID-19 pandemic on the medical care and health-care behaviour of patients with lupus and other systemic autoimmune diseases: a mixed methods longitudinal study. Rheumatol Advances Pract.

[CR47] Tucker JD, Wong B, Nie J-B, Kleinman A (2016). Rebuilding patient–physician trust in China. Lancet.

[CR48] Udow-Phillips M, Lantz PM (2020). Trust in Public Health is Essential amid the COVID-19 pandemic. J Hosp Med.

[CR49] United Nations (2021) Standard country or area codes for statistical use (M49)

[CR50] Vinck P, Pham PN, Bindu KK (2019). Institutional trust and misinformation in the response to the 2018–19 Ebola outbreak in North Kivu, DR Congo: a population-based survey. Lancet Infect Dis.

[CR51] Wong MCS, Wong ELY, Huang J (2021). Acceptance of the COVID-19 vaccine based on the health belief model: a population-based survey in Hong Kong. Vaccine.

[CR52] Zhao D, Zhao H, Cleary PD (2019). International variations in trust in health care systems. Int J Health Plann Mgmt.

[CR53] Zhao D, Zhao H, Cleary PD (2019). Understanding the determinants of public trust in the health care system in China: an analysis of a cross-sectional survey. J Health Serv Res Policy.

