# Supplementary material for: Trust in Healthcare during COVID-19 in Europe: vulnerable groups trust the least
Source: Z Gesundh Wiss. 2022 Mar 24:1–10. Online ahead of print. doi: 10.1007/s10389-022-01705-3 (PMC8944407; doi:10.1007/s10389-022-01705-3)

*Figure A1*. *Results from Linear Regression Analyses Predicting Trust in the Healthcare System During COVID-19 within European Countries.*
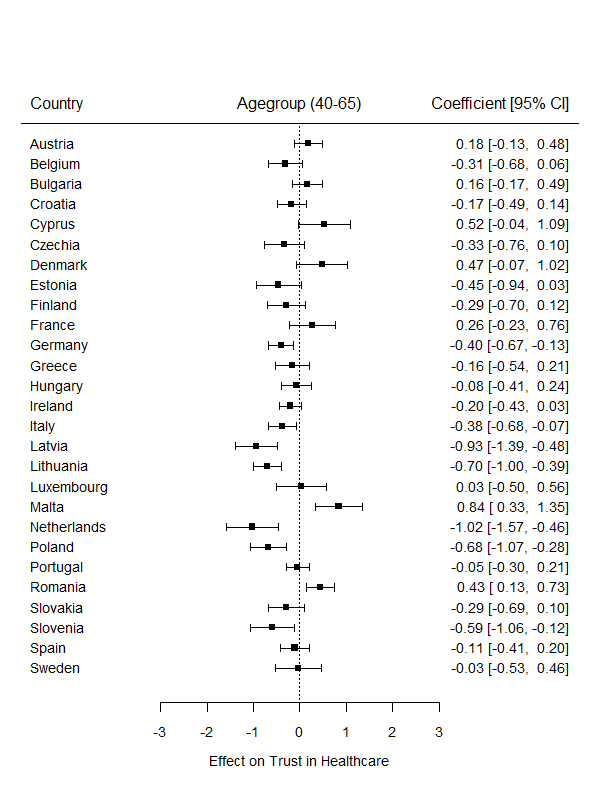

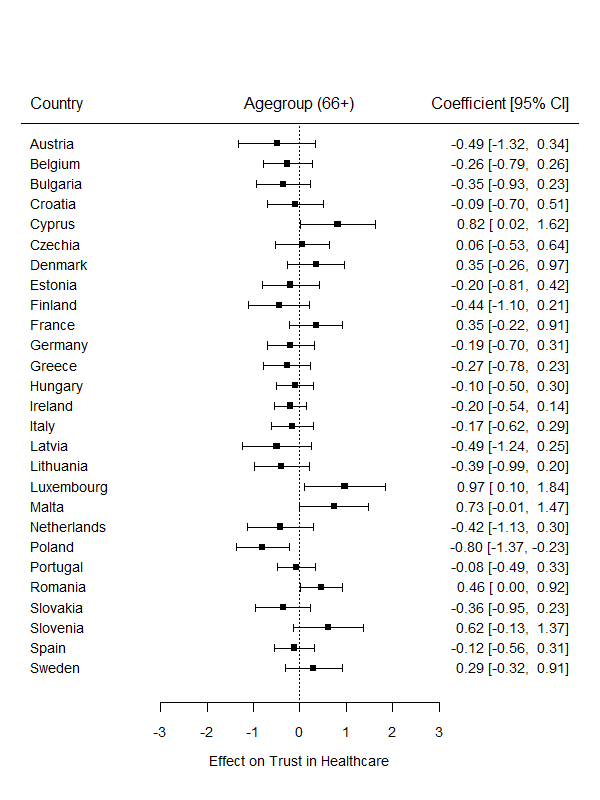


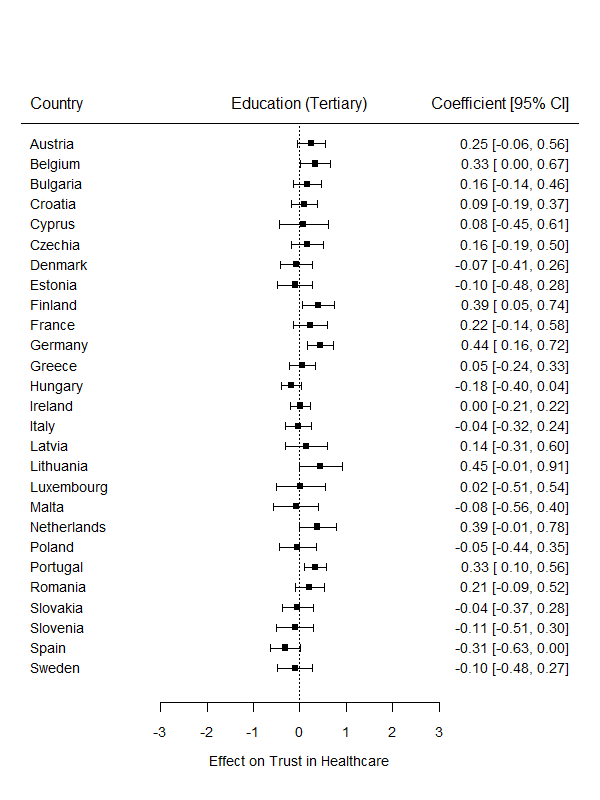

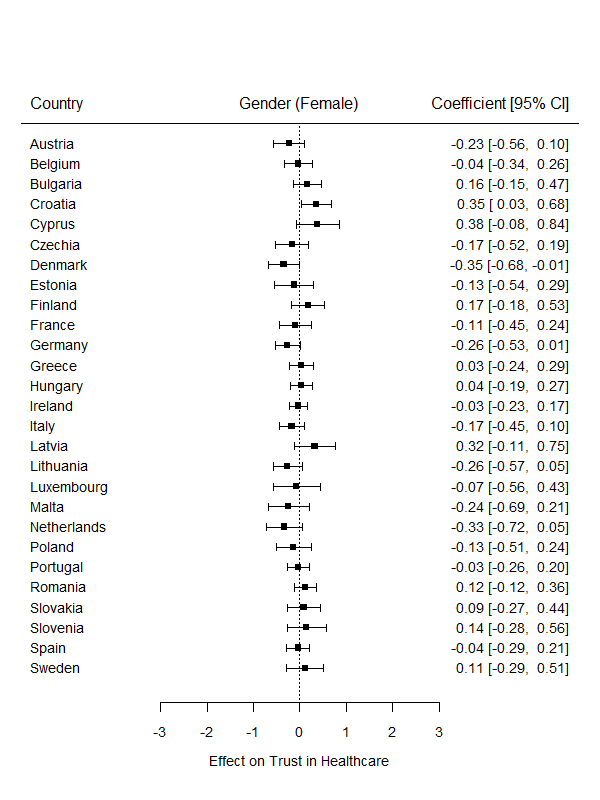

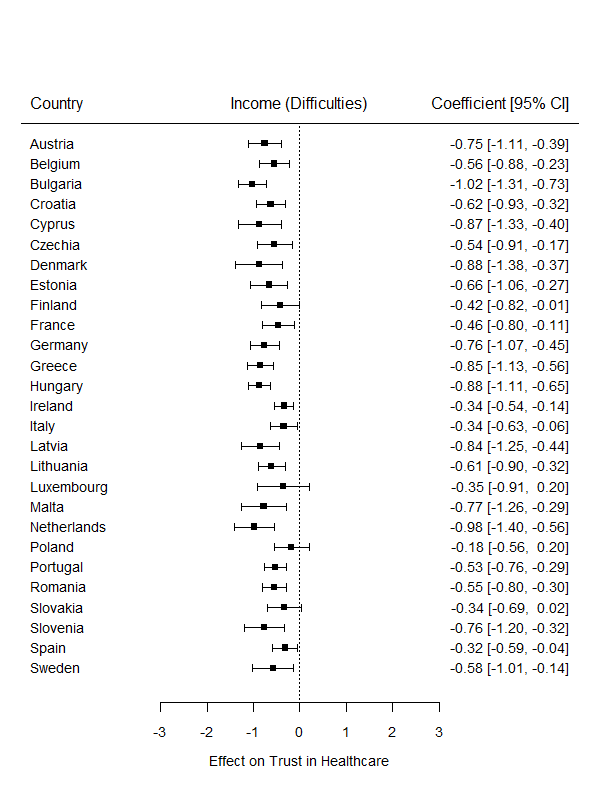

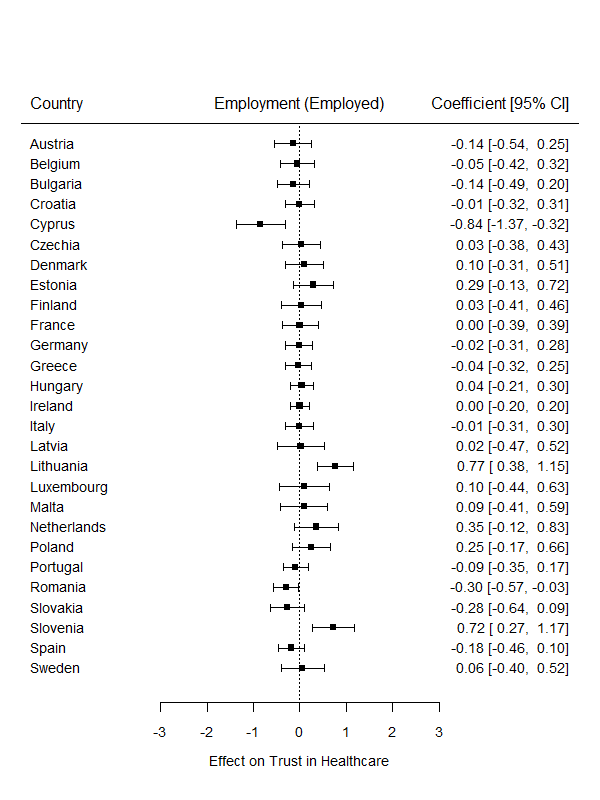

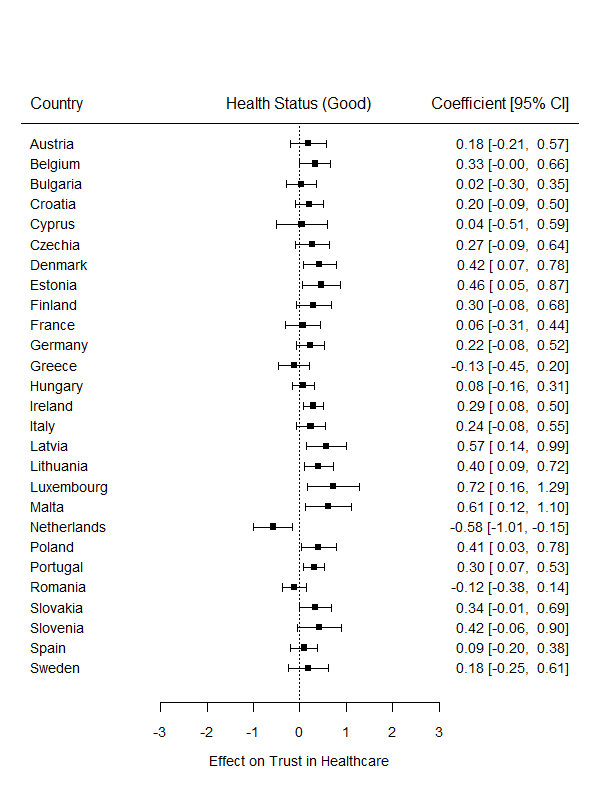

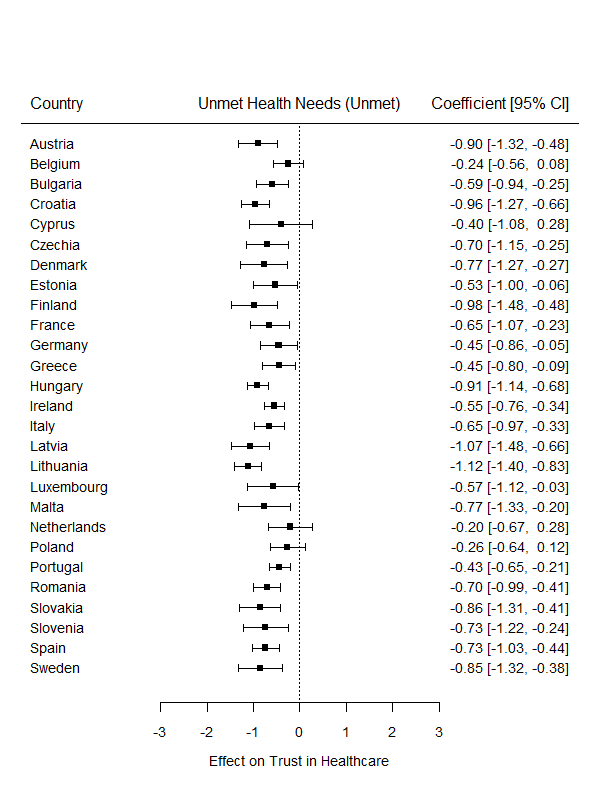

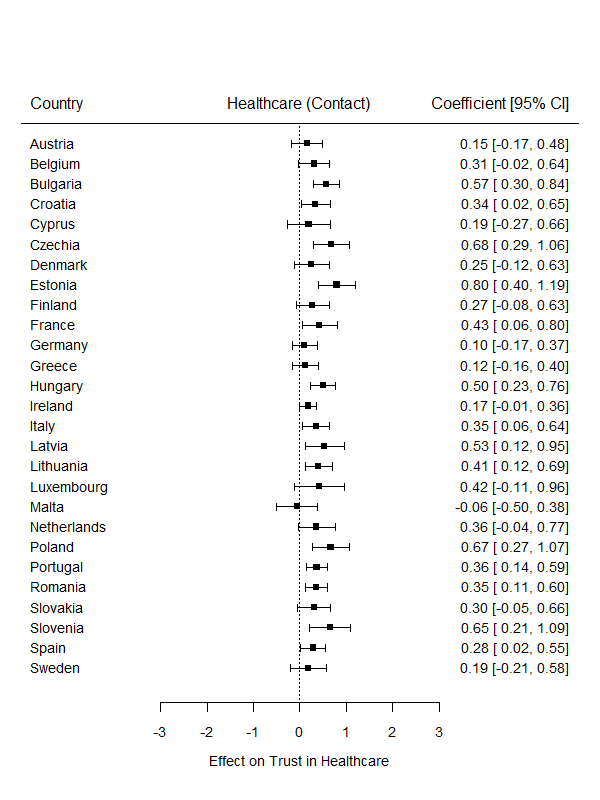

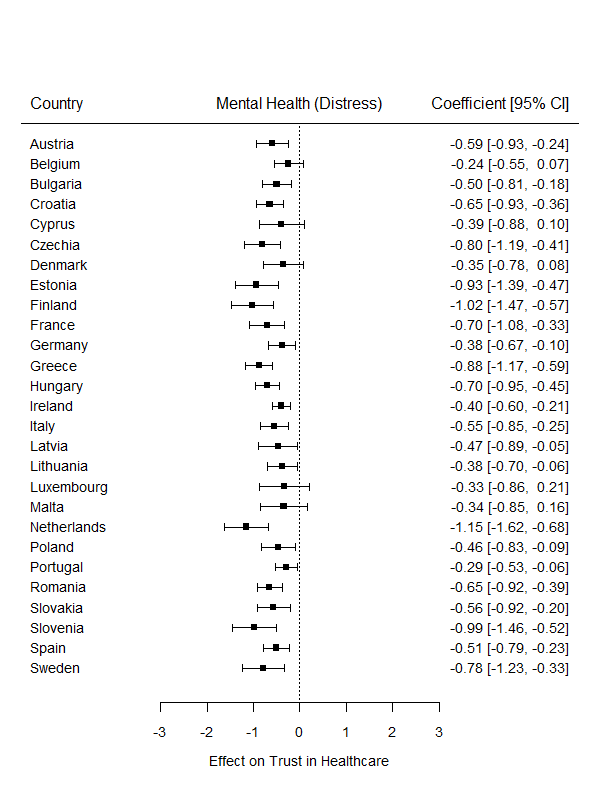

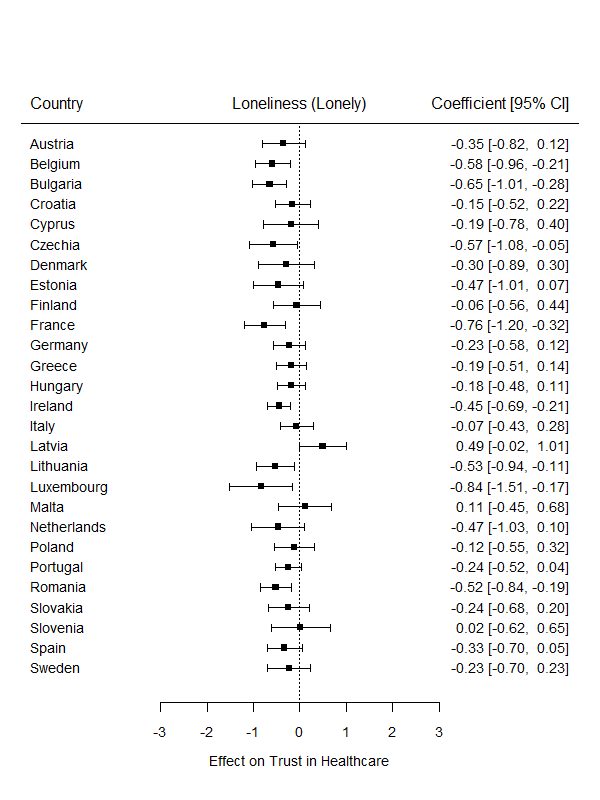

Supplement: Supplementary file 1 — (DOCX 212 kb) [file 10389_2022_1705_MOESM1_ESM.docx]
